# Trait Sensitivity to Negative and Positive Feedback Does Not Interact With the Effects of Acute Antidepressant Treatment on Hedonic Status in Rats

**DOI:** 10.3389/fnbeh.2020.00147

**Published:** 2020-08-27

**Authors:** Paulina Surowka, Karolina Noworyta, Rafal Rygula

**Affiliations:** Affective Cognitive Neuroscience Laboratory, Department of Pharmacology, Maj Institute of Pharmacology Polish Academy of Sciences, Krakow, Poland

**Keywords:** feedback sensitivity, cognitive bias, anhedonia, animal model, antidepressant

## Abstract

Aberrant cognition plays a pivotal role in the development and maintenance of depression. One of the most important cognitive distortions associated with depression is aberrant sensitivity to performance feedback. Under clinical conditions, this sensitivity can be measured using the probabilistic reversal learning (PRL) test, which has also been recently implemented in animal studies. Although the evidence for the coexistence of depression and altered feedback sensitivity is relatively coherent, it is unclear whether this sensitivity can influence the effectiveness of antidepressant treatment. In the present research, we investigated how trait sensitivity to negative and positive feedback interacts with the effects of acute antidepressant treatment on hedonic status in rats. We tested a cohort of rats with a series of 10 PRL tests, and based on this screening, we classified each animal as sensitive or insensitive to negative and positive feedback. Subsequently, in the Latin square design, we evaluated the effects of a single administration of two antidepressant drugs (each at three different doses: agomelatine: 5, 10, and 40 mg/kg; mirtazapine 0.5, 1, and 3 mg/kg) on the hedonic status of rats in the sucrose preference tests. There was no statistically significant interaction between trait sensitivity to feedback and the effects of acute antidepressant treatment on hedonic status in rats.

## Introduction

A depressive disorder is a serious mental illness characterized by lowered mood and anhedonia (i.e., the loss of pleasure; Belzung et al., 2015). It has also been associated with sustained widespread cognitive impairments, including abnormal responses to negative (NF) and positive (PF) feedback (Clark et al., 2009). Indeed, several studies have demonstrated that depressed individuals are hypersensitive to NF (punishments) and hyposensitive to PF (rewards), which leads to altered processing of negatively and positively valenced information (Beats et al., 1996; Elliott et al., 1998). Such negatively distorted thinking perpetuates a maladaptive belief system and low mood, leading to a specific state in which criticism or minor errors are overemphasized and major achievements are ignored (Clark et al., 2009). Although clinical evidence for the coexistence of depression and altered sensitivity to performance feedback is relatively coherent, we still do not know whether increased or decreased sensitivity to NF/PF are associated with better/worse antidepressant treatment outcomes.

One of the most influential, recent theories of the antidepressant drug action, implies that antidepressants may produce their ultimate, clinical effects by early actions on information processing biases, including distorted sensitivity to feedback (Harmer et al., 2003b, 2009). It has been proposed that, at a neuropsychological level, antidepressant drugs remediate the negative affective biases and that, contrary to common opinion, these actions occur relatively quickly, even following a single drug administration (Harmer et al., 2009). This induction of a more positive way of processing environmental stimuli (positive bias) leads to cognitive and psychological reconsolidation and wider antidepressant effect. There is now a growing body of experimental evidence that antidepressants can affect emotional processing very early in treatment. Several studies revealed that in healthy subjects, even single doses of antidepressants increase the recognition of happy facial expressions (Harmer et al., 2003a,b), and increase attention to positive, socially relevant stimuli in a visual probe task (Browning et al., 2007). Studies in animals yielded similar results. It has been demonstrated that acute administration of several widely prescribed antidepressants changes the affective bias of naïve rats in the affective bias test [citalopram, desipramine, fluoxetine, mirtazapine, venlafaxine, reboxetine, clomipramine and ketamine (Stuart et al., 2013, 2015)], modulate cognitive judgment bias in the ambiguous-cue interpretation (ACI) test [citalopram, desipramine (Rygula et al., 2014; Golebiowska and Rygula, 2017) and reboxetine (Enkel et al., 2010; Anderson et al., 2013)], and alter the sensitivity of rats to performance feedback in the preclinical version of the PRL task [agomelatine, mirtazapine (Drozd et al., 2019), citalopram (Bari et al., 2010) and ketamine (Rychlik et al., 2017)].

The recent implementation of the preclinical version of the probabilistic reversal learning (PRL) paradigm (Bari et al., 2010) allowed for the investigation of this question in animal models. The results of our previous studies demonstrated that in rodents, sensitivity to NF and PF are stable and enduring behavioral traits (Noworyta-Sokolowska et al., 2019) and that even single doses of agomelatine or mirtazapine could change this sensitivity in the PRL test (Drozd et al., 2019). In that latter study, acute agomelatine treatment reduced the sensitivity of rats to NF, as indexed by the decreased proportion of lose-shift behaviors, while mirtazapine increased the sensitivity of rats to PF, as indexed by the increased proportion of win-stay behaviors. This decrease in NF sensitivity and the increased sensitivity to PF were hypothesized to manifest antidepressant-induced, positive, information-processing biases, similar to those reported previously in humans following acute antidepressant treatment (Arnone et al., 2009; Rawlings et al., 2010; Komulainen et al., 2016).

In the current study, we build off of these prospective findings by testing a hypothesis that the effects of antidepressant drugs on reward-related processes may be influenced by trait sensitivity to NF or PF. In other words, we designed this study to investigate whether trait sensitivity to NF/PF could boost/diminish the effects of acute antidepressant treatment on the hedonic capacity of rats.

The animals were screened in a series of PRL tests and classified as sensitive/insensitive to NF/PF. Subsequently, the influence of this trait on the effects of acute administration of two antidepressant drugs, namely, mirtazapine and agomelatine (each in three different doses), on the hedonic processing of rats was investigated using sucrose preference (SP) tests.

## Materials and Methods

### Subjects and Housing

In the present study, we used 80 male Sprague–Dawley rats (Charles River, Germany) weighing 175–200 g (about 10 weeks old) upon arrival. Rats were kept in groups (four animals/cage) under controlled temperature (21 ± 1°C) and humidity (40–50%) under a 12/12 h light/dark cycle (lights on at 7:00 h). The cage size was 56 (L) × 35 (W) × 21 (H) cm.

During the entire experiment, rats were mildly food restricted to 85% of their free-feeding weight (according to normal growth curve recommended by the laboratory rodent supplier—Charles River Research Models and Services Catalogue) by providing 15–20 g of food pellets per rat per day (standard laboratory chow). Food restriction began 1 week before behavioral training. Water was available *ad libitum*.

The experiments were performed during the light phase of the light/dark cycle.

### Apparatus

The PRL training and testing was performed in 16 computer-controlled operant conditioning boxes (Med Associates, St. Albans, Vermont, VT, USA). Boxes were equipped with a fan, light, speaker, a food dispenser set to deliver a sucrose pellet (Dustless Precision Pellets, 45 mg; Bio-Serv, Flemington, NJ, USA), and two retractable levers which were located on opposite sides of the feeder. We have programmed the experimental protocols using Med State notation code (Med Associates). The data were analyzed using a custom-written R programme. The experimental procedure for the PRL task used in this study was a modified version of the procedures used and described previously by Bari et al. (2010) and has been described in detail elsewhere (Noworyta-Sokolowska et al., 2019).

### Measuring Feedback Sensitivity Using the PRL Test

#### PRL Training and Testing

After the initial instrumental training described elsewhere (Noworyta-Sokolowska et al., 2019), the rats were trained in the PRL paradigm. Each PRL training session consisted of 200 trials, and each trial lasted for a maximum of 22 s. The start of a trial was signalled by the house light, which remained on until the end of the trial. Two seconds after the trial had started, both levers were presented, and one of them was randomly assigned as the “correct” lever, which delivered a reward 80% of the times it was pressed. A press on the other lever—the “incorrect” lever—would result in a rewarding outcome only 20% of the times it was pressed. No response in 10 s triggered the ITI and was counted as an omission. The same ITI directly followed a punishing outcome, i.e., no reward on 20% of the “correct” and 80% of the “incorrect” lever presses. After every eight consecutive “correct” lever presses (regardless of the outcome), the criterion for the reversal of the outcome probabilities was reached. The previously “correct” lever now became “incorrect” and vice versa. This pattern was followed until the end of the session.

This training phase was repeated daily until the individual animals achieved sufficient performance levels. The criteria to be met were a minimum of three reversals completed during three consecutive training sessions, with less than 15% omissions per session.

#### Parameters Measured in the PRL Test

To monitor the sensitivity of rats to PF and NF, the animals’ decisions were tracked on a trial-by-trial basis. To evaluate the sensitivity to NF we assessed the ability of animals to ignore infrequent and misleading, punished (non-rewarded) outcomes on the “correct” lever. For this, the animal’s decisions to switch levers following such a misleading punishment (probabilistic lose-shifts), were scored and expressed as a ratio of all punished (unrewarded) outcomes on that lever. To evaluate sensitivity to PF, all rewarded outcomes (true and misleading) followed by a decision to stay with the lever that delivered them (win-stays) were counted jointly for the “correct” and “incorrect” levers and expressed as a ratio of all rewarded outcomes.

### Measuring Hedonic Capacity Using the SP Test

The preference for palatable sweet solutions is the most frequently used test to measure sensitivity to rewards/hedonic capacity in rodents (Papp et al., 1991; Willner et al., 1992). In this test, animals can choose between a palatable sweet solution and plain water, and the decreased or increased preference for the palatable solution is considered to reflect the decrease or increase in hedonic capacity respectively. The advantages of this test, which explain its popularity in laboratories throughout the world, are its simplicity and reliability. The method itself has been used in our laboratory for several years and has been thoroughly validated using various behavioral and pharmacological manipulations (Rygula et al., 2005, 2006, 2008, 2013; Noworyta-Sokolowska et al., 2019). During the SP test, the rats were separated into single cages and were offered a voluntary choice between two bottles for 1 h, where one bottle contained a 2% (w/v) sucrose solution and the other bottle contained tap water. To prevent potential effects of side preference in drinking, the position of the bottles was switched after 30 min. The consumption of water and sucrose solution was measured by weighing the bottles. The preference for sucrose was calculated from the amount of sucrose solution consumed and is expressed as a percentage of the total amount of liquid that was consumed.

### Experimental Design and Drugs

**Figure 1:**
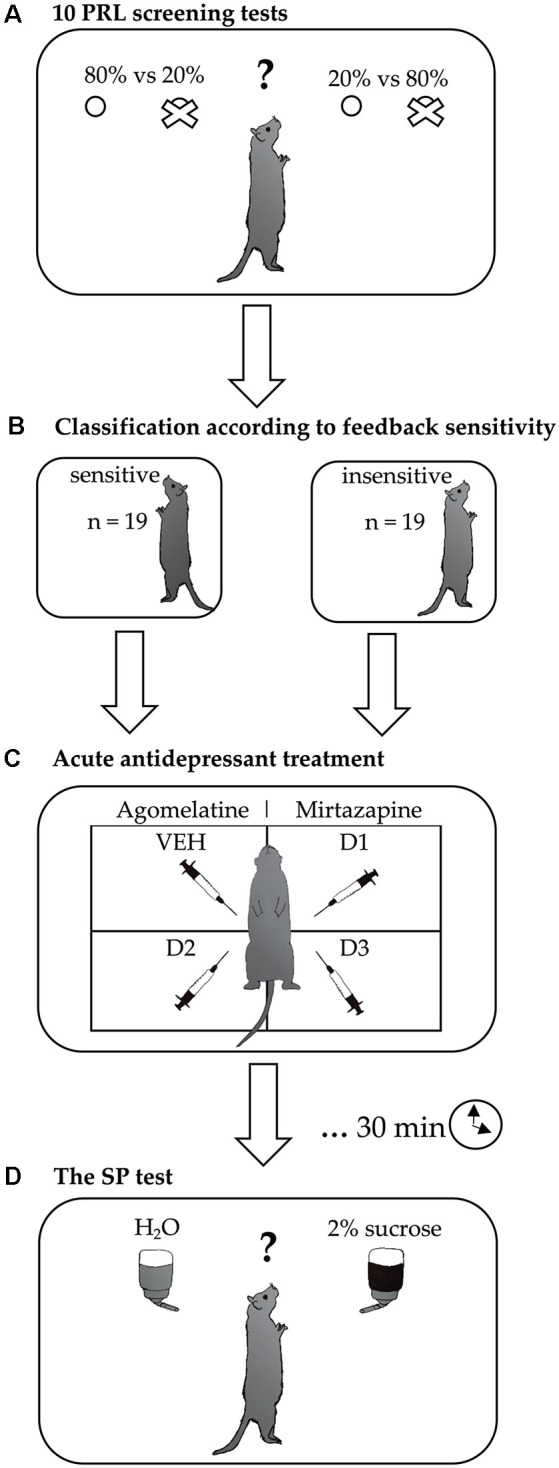
Experimental design. After achieving a stable performance in the probabilistic reversal learning (PRL) training sessions, the rats were subjected to **(A)** feedback-sensitivity screening, consisting of 10 PRL tests carried out over 10 consecutive days. Based on the results of this screening, each rat was **(B)** classified as insensitive or sensitive to negative and positive feedback. Subsequently, differences in the effects of **(C)** acute treatment with two different antidepressant drugs (agomelatine and mirtazapine, each in three doses) on hedonic processing were evaluated using **(D)** sucrose preference (SP) tests conducted in a fully randomized Latin square design.

The experimental schedule is presented in Figure 1. Initially, the rats were trained for the PRL test as described above. After achieving a stable performance, animals that reached the criterion were subsequently tested in 10 consecutive PRL tests over 10 days. Based on this “sensitivity screening,” the rats were divided using a median split into sensitive and insensitive to NF and PF. The division according to sensitivity to NF was made based on the average ratio of lever changes following misleading punishment (probabilistic lose-shifts) made by the animals across all 10 screening tests. The division according to the sensitivity to PF was made based on the average ratio of pressing the same lever (win-stays) following both true and misleading rewards across all 10 screening tests. To confirm the stability of the feedback sensitivity traits, we additionally analyzed the “frequency of sensitivity,” expressed as the number of the PRL tests (out of the 10 comprising screening) in which an animal displayed sensitivity to feedback. After the feedback sensitivity screening, the effects of acute administration of agomelatine and mirtazapine on the hedonic capacity of rats were evaluated using SP tests in the fully randomized Latin square design, which means that on any given day all treatments were represented and were balanced across the tests. This within-subject study design, contrary to the between-subject designs, allowed us to reduce the variance of the data and the number of animals used. The use of a Latin square design is a common and valid method in pharmacological research (Howell, 1997). It has been also successfully applied in a number of our previous studies (Drozd et al., 2017, 2019; Golebiowska and Rygula, 2017; Rychlik et al., 2017).

The drugs and their doses were chosen based upon our previous study, which demonstrated that even single doses of agomelatine or mirtazapine could change the sensitivity to the feedback of experimental animals in the PRL test (Drozd et al., 2019). Agomelatine (TCI Europe, Zwijndrecht, Belgium, HPLC −98%), a relatively novel antidepressant drug that acts as a potent agonist of melatonin MT1/MT2 receptors (Yous et al., 1992; Ying et al., 1996) and an antagonist of the 5-HT2C receptor subtype (Millan et al., 2003) was dissolved in 1% hydroxyethyl cellulose and applied in doses 5, 10, and 40 mg/kg). Mirtazapine (TCI Europe, Zwijndrecht, Belgium, HPLC −98%), a tetracyclic antidepressant modulating noradrenergic and serotonergic neurotransmission *via* blockade of central α2-adrenergic auto- and heteroreceptors, stimulation of 5-HT1A receptors (Berendsen and Broekkamp, 1997), and blockade of 5-HT2A, 5-HT2C and 5-HT3 receptors (de Boer, 1995), was dissolved in an equimolar solution of citric acid and injected in doses 0.5, 1, and 3 mg/kg. The drugs were administered intraperitoneally in a dose volume of 1 ml/kg 30 min. before the SP test. Control animals received corresponding injections of vehicle solutions. The wash-out period between administrations of different drug doses in the Latin square design was 1 week, what considering the pharmacokinetics of tested drugs (the elimination half-lives of a few hours), was more than enough to avoid the accumulation of drug effects (Zupancic and Guilleminault, 2006; Rouini et al., 2014; He et al., 2018).

### Statistics

The data were analyzed using SPSS (version 25.0, SPSS Inc., Chicago, IL, USA). The distribution of the experimental data was tested using the Kolmogorov–Smirnov test. The effects of trait sensitivity to NF/PF and the effects of antidepressant treatment on parameters measured in the SP test were investigated using two-way repeated-measures ANOVAs with the between-subject factor of sensitivity (two levels: “sensitive,” “insensitive”) and the within-subject factor of dose (four levels: vehicle, D1, D2, and D3). Homogeneity of variance and sphericity of ANOVA were verified using Levene’s and Mauchly’s tests, respectively. For pairwise comparisons, the values were adjusted using the Sidak correction (Howell, 1997). All of the tests of significance were performed at *α* = 0.05.

## Results

### Effects of Acute Agomelatine Administration on Sucrose Preference in Rats Classified as Sensitive/Insensitive to Negative and Positive Feedback

All animals fulfilled the training criteria and qualified for PRL screening. Two rats were removed from the analysis due to fluid leakage during the SP test.

#### Negative Feedback Sensitivity Screening

**Figure 2:**
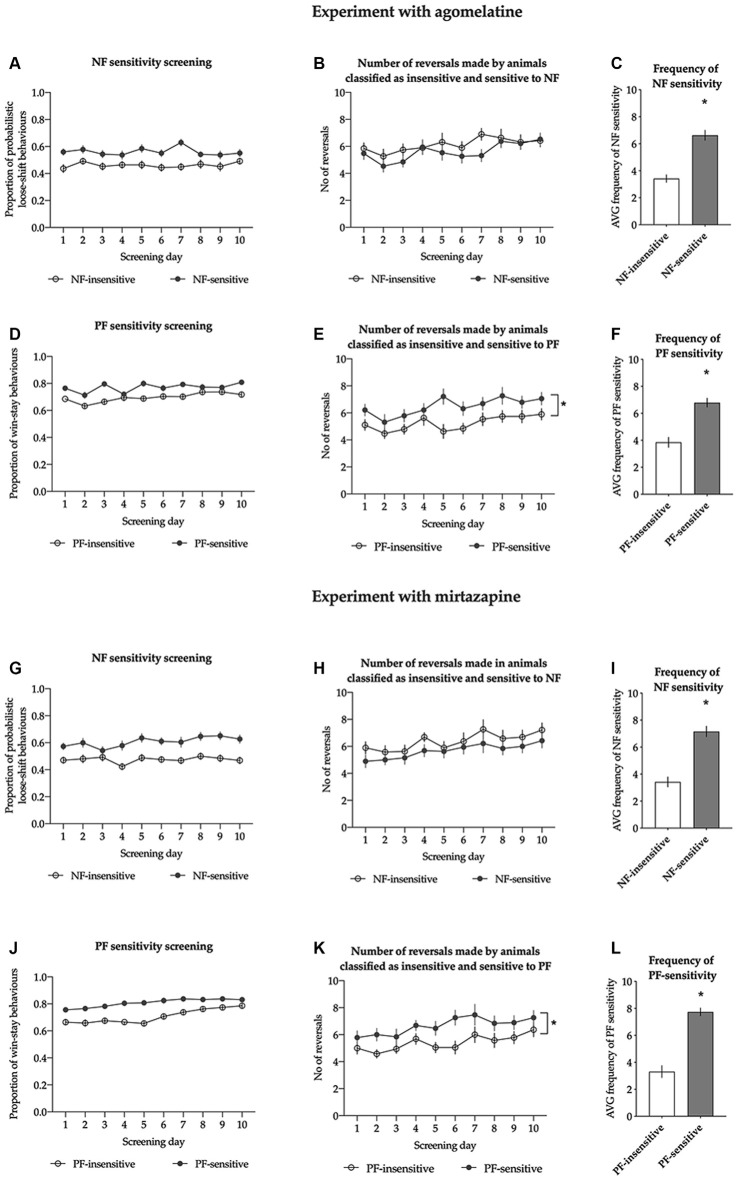
Results of the sensitivity to feedback screening in animals treated with agomelatine **(A–F)** and mirtazapine **(G–L)**. Panels **(A,G)** show the average proportion of lose-shift behaviors following misleading punishment in rats classified as insensitive (open circles, *N* = 19) and sensitive (filled circles, *N* = 19) to negative feedback (NF) across all 10 screening probabilistic reversal learning (PRL) tests in cohorts of rats treated with agomelatine and mirtazapine, respectively. Panels **(B,H)** show the average number of reversals in rats classified as insensitive (open circles, *N* = 19) and sensitive (filled circles, *N* = 19) to NF across all 10 screening PRL tests in cohorts of rats treated with agomelatine and mirtazapine, respectively. Panels **(C,I)** show the average frequency of sensitivity to NF in a cohort of rats treated with agomelatine and mirtazapine respectively. Panels **(D,J)** show the average proportion of win-stay behaviors following a reward in rats classified as insensitive (open circles, *N* = 19) and sensitive (filled circles, *N* = 19) to positive feedback (PF) across all 10 screening PRL tests. Panels **(E,K)** show the average number of reversals in rats classified as insensitive (open circles, *N* = 19) and sensitive (filled circles, *N* = 19) to PF across all 10 screening PRL tests in cohorts of rats treated with agomelatine and mirtazapine, respectively. Panels **(F,L)** show the average frequency of sensitivity to PF in a cohort of rats treated with agomelatine and mirtazapine respectively. The frequency is expressed as the number of PRL tests (out of the 10 comprising screening) in which an animal displayed the value of given feedback sensitivity located above the median of the values from the entire cohort. Data are presented as the mean ± SEM. *Indicates *p* < 0.05 compared to the insensitive group.

For the animals classified as NF-insensitive, the average proportion of lose-shift behaviors following misleading NF ranged from 0.341 to 0.501, with an average of 0.461 ± 0.009. For those classified as NF-sensitive, the average proportion of probabilistic lose-shift behaviors ranged from 0.503 to 0.734 with an average of 0.561 ± 0.013. The sensitivity to NF in both subgroups was stable across the screening period (nonsignificant Screening day × NF sensitivity interaction (*F*_(9,324)_ = 0.913, *p* = 0.514, Figure 2A).

The average number of reversals made by the animals classified as insensitive to NF during the screening period ranged from 4.6 to 9.5, with an average of 6.12 ± 0.28; for animals classified as sensitive to NF, the average number of reversals ranged from 3.6 to 7.7, with an average of 5.60 ± 0.24. Reversal performance in both groups was stable [there was a nonsignificant interaction between screening day and NF sensitivity (*F*_(9,324)_ = 0.662, *p* = 0.743), Figure 2B].

The average frequency of NF sensitivity in animals classified as NF-insensitive ranged from 0 to 5, with an average of 3.4 ± 0.3; in those classified as NF sensitive, the average frequency of NF sensitivity ranged from 5 to 10, with an average of 6.6 ± 0.4. The animals classified as NF-insensitive were significantly less sensitive to NF than the rats classified as NF-sensitive (*t* = 6.853, *df* = 36, *p* < 0.001, Figure 2C).

#### Positive Feedback Sensitivity Screening

The average proportion of win-stay behaviors in the animals classified as PF-insensitive ranged from 0.635 to 0.725, with an average of 0.696 ± 0.006. The average proportion of win-stay behaviors in the animals classified as PF-sensitive ranged from 0.733 to 0.865, with an average of 0.770 ± 0.008. The sensitivity to PF in both subgroups was stable across the screening period [there was a nonsignificant interaction between screening day and PF sensitivity (*F*_(9,324) =_1.740, *p* = 0.079, Figure 2D)].

The average number of reversals made by the animals classified as insensitive to PF during the screening period ranged from 3.6 to 6.7, with an average of 5.24 ± 0.19. This average was significantly (*p* < 0.05) lower than that for animals classified as sensitive to PF, where it ranged from 4.6 to 9.5 with an average of 6.48 ± 0.26. Reversal performance in both groups was stable [there was a nonsignificant interaction between screening day and PF sensitivity (*F*_(9,324)_ = 0.740, *p* = 0.672, Figure 2E)].

The average frequency of PF sensitivity in animals classified as PF-insensitive ranged from 1 to 7, with an average of 3.8 ± 0.4; in those classified as PF-sensitive, the average frequency of PF sensitivity ranged from 5 to 10, with an average of 6.8 ± 0.3. The animals classified as PF-insensitive were statistically significantly less sensitive to PF than those classified as PF-sensitive (*t* = 5.958, *df* = 36, *p* < 0.001, Figure 2F).

#### Sucrose Preference Test

**Figure 3:**
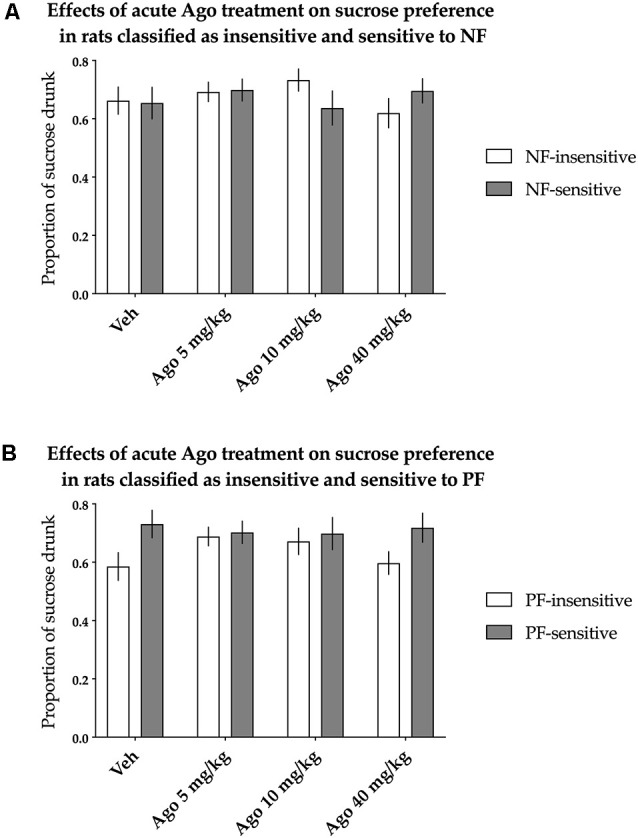
Effects of acute administration of agomelatine (Ago) on the hedonic status of rats measured in the sucrose preference test. The data represent the average sucrose preference of rats classified as insensitive (open bars) and sensitive (filled bars) to **(A)** negative feedback (NF) and **(B)** positive feedback (PF) following acute administration of three different doses (5, 10 and 40 mg/kg) of agomelatine and vehicle solution. Data are presented as the mean ± SEM. *N* = 19 rats per group.

The animals classified as NF-insensitive and NF-sensitive did not differ in sucrose preference either basally or after acute treatment with agomelatine [nonsignificant effect of NF sensitivity (*F*_(1,36)_ = 0.014, *p* = 0.907) and not significant Treatment × NF sensitivity interaction (*F*_(3,108)_ = 1.733, *p* = 0.164)]. Acute agomelatine treatment itself also had no statistically significant effects on sucrose preference [there was a nonsignificant effect of treatment (*F*_(3,108)_ = 0.504, *p* = 0.680), Figure 3A].

Similarly, PF sensitivity had no statistically significant effects on sucrose preference either basally or after acute treatment with agomelatine [the effect of PF sensitivity was nonsignificant (*F*_(1,36)_ = 3.181, *p* = 0.083), and there was a nonsignificant interaction between treatment and PF sensitivity (*F*_(3,108)_ = 1.500, *p* = 0.219, Figure 3B)].

### Effects of Acute Mirtazapine Administration on Sucrose Preference in Rats Classified as Sensitive/Insensitive to Negative and Positive Feedback

All animals fulfilled the training criteria and qualified for PRL screening. Two rats were removed from the analysis due to fluid leakage during the SP test.

#### Negative Feedback Sensitivity Screening

For the animals classified as NF-insensitive, the proportion of lose-shift behaviors following misleading NF ranged from 0.390 to 0.518, with an average of 0.475 ± 0.008. For those classified as NF-sensitive, the proportion of probabilistic lose-shift behaviors ranged from 0.521 to 0.722, with an average of 0.607 ± 0.015. The sensitivity to NF in both subgroups was stable across the screening period [there was a nonsignificant screening day × NF sensitivity interaction (*F*_(9,324) =_0.833, *p* = 0.586, Figure 2G)].

The number of reversals made by the animals classified as NF-insensitive during the screening period ranged from 3.5 to 9.9, with an average of 6.38 ± 0.33; for animals classified as NF-sensitive, the number of reversals ranged from 3.8 to 8.1, with an average of 5.68 ± 0.24. Reversal performance in both groups was stable [there was a nonsignificant interaction between screening day and NF sensitivity (*F*_(9,324)_ = 0.174, *p* = 0.997, Figure 2H)].

The frequency of NF sensitivity in animals classified as NF-insensitive ranged from 0 to 7, with an average of 3.4 ± 0.4; in those classified as NF-sensitive, the frequency of NF sensitivity ranged from 5 to 10, with an average of 7.16 ± 0.38. The animals classified as NF-insensitive were statistically significantly less sensitive to NF than the rats classified as NF-sensitive (*t* = 7.011, *df* = 36, *p* < 0.001, Figure 2I).

#### Positive Feedback Sensitivity Screening

The proportion of win-stay behaviors in the animals classified as PF-insensitive ranged from 0.649 to 0.755, with an average of 0.708 ± 0.007. The proportion of win-stay behaviors in the animals classified as PF-sensitive ranged from 0.759 to 0.875, with an average of 0.808 ± 0.007. The sensitivity to PF in both subgroups was stable across the screening period [there was a nonsignificant screening day × PF sensitivity interaction (*F*_(9,324) =_1.726, *p* = 0.082, Figure 2J)].

The number of reversals made by the animals classified as PF-insensitive during the screening period ranged from 3.5 to 7.1, with an average of 5.41 ± 0.21. This average was significantly (*p* < 0.05) lower than that for animals classified as PF-sensitive, where it ranged from 4.3 to 9.9 with an average of 6.65 ± 0.30. Reversal performance in both groups was stable [there was a nonsignificant interaction between screening day and PF sensitivity (*F*_(9,324)_ = 0.427, *p* = 0.920, Figure 2K)].

The frequency of sensitivity to PF in animals classified as PF-insensitive ranged from 0 to 7, with an average of 3.3 ± 0.4; in those classified as PF-sensitive, the frequency of sensitivity ranged from 6 to 10, with an average of 7.7 ± 0.3. The animals classified as PF-insensitive were statistically significantly less sensitive to PF than those classified as PF-sensitive (*t* = 8.268, *df* = 36, *p* < 0.001, Figure 2L).

#### Sucrose Preference Test

**Figure 4:**
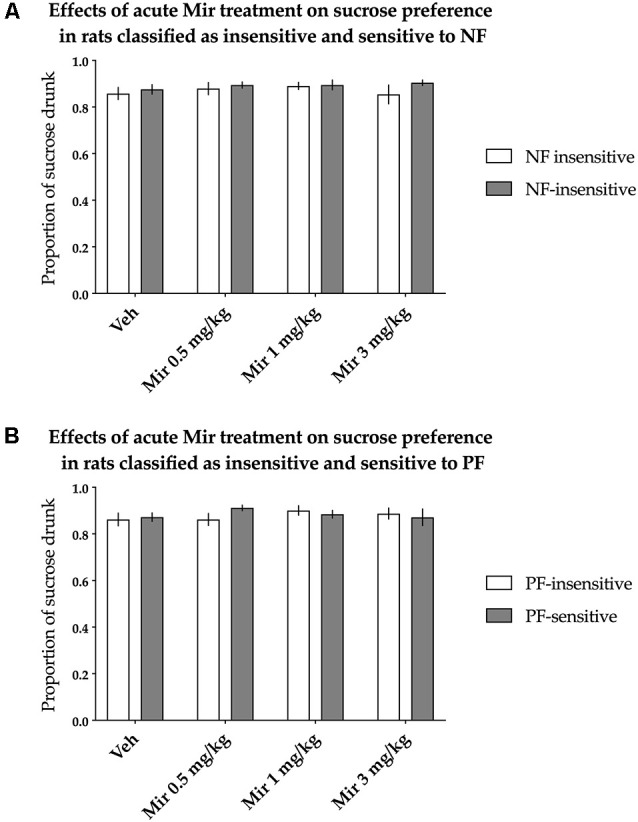
Effects of acute administration of mirtazapine (Mir) on the hedonic status of rats, measured in the sucrose preference test. The data represent the average sucrose preference of rats classified as insensitive (open bars) and sensitive (filled bars) to **(A)** negative feedback (NF) and **(B)** positive feedback (PF) following acute administration of three different doses of mirtazapine (0.5, 1 and 3 mg/kg) and vehicle solution. Data are presented as the mean ± SEM. *N* = 19 rats per group.

The animals classified as NF-insensitive and NF-sensitive did not differ in sucrose preference either basally or after acute treatment with mirtazapine [nonsignificant effect of NF sensitivity (*F*_(1,36)_ = 0.883, *p* = 0.354) and nonsignificant treatment × NF sensitivity interaction (*F*_(3,108)_ = 0.514, *p* = 0.674)]. Acute mirtazapine treatment itself also had no statistically significant effects on sucrose preference [nonsignificant effect of treatment (*F*_(3,108)_ = 0.645, *p* = 0.588, Figure 4A)].

Similarly, PF sensitivity had no statistically significant effects on sucrose preference either basally or after acute treatment with mirtazapine [nonsignificant effect of PF sensitivity (*F*_(1,36)_ = 0.088, *p* = 0.769) and nonsignificant treatment × PF sensitivity interaction (*F*_(3,108)_ = 1.302, *p* = 0.278, Figure 4B)].

## Discussion

The results presented here confirmed that in rats, sensitivity to NF and PF can be considered stable and enduring behavioral traits. They also confirmed that these traits do not interact with basal hedonic capacity. Most importantly, they demonstrated that trait sensitivity to feedback does not determine the effects of acute administration of two antidepressant drugs, agomelatine and mirtazapine, on hedonic processing in rats.

Over the past years, behavioral research has revealed that a concept of cognitive/behavioral traits exists and can be measured in animals (Gosling, 2001). It also revealed that there exists considerable cross-species overlap for some of these traits and that the assessment of these traits in animals has numerous practical applications that can contribute to a better understanding of psychiatric disorders (Rygula et al., 2018). For instance, recent studies using animal models have demonstrated that trait “pessimism,” which has been previously linked with increased sensitivity to NF (Rygula and Popik, 2016), is associated with a “pro-depressive profile” that predicts increased vulnerability to stress-induced anhedonia (Rygula et al., 2013) and motivational deficits (Drozd et al., 2017). They also showed that biased judgement, as a trait, is associated with alterations in the effectivity of antidepressant drug treatment (Drozd et al., 2019). Other studies suggested that trait sensitivity to NF and/or PF could be candidates for a cognitive biomarker of depression (Noworyta-Sokolowska et al., 2019). Although we did not observe statistically significant interactions between trait sensitivity to feedback and the hedonic capacity of tested animals in the present study, this result was not surprising and has already been explained elsewhere (Noworyta-Sokolowska et al., 2019) using Beck’s cognitive model of depression (Beck, 1987, 2008). According to this theory, although biased acquisition and processing of information has a primary role in the maintenance and recurrence of depression, the development of depressive symptoms usually requires environmental triggers, e.g., stress. Thus, studies using animal models of depression based on chronic stress will be required to ultimately confirm whether the sensitivity to feedback (especially to PF) is a latent trait that could determine the hedonic capacity of rats.

It has also been recently proposed that antidepressant drugs may produce their ultimate clinical effects by early actions on information processing biases (Harmer et al., 2003b, 2009). Indeed, in our previous study, we demonstrated that both agomelatine and mirtazapine produce rapid effects on feedback sensitivity in the PRL paradigm (Drozd et al., 2019). In that study, acute agomelatine treatment reduced the sensitivity of rats to NF, as indexed by the decreased proportion of lose-shift behaviors, while mirtazapine increased the sensitivity of rats to PF, as indexed by the increased proportion of win-stay behaviors. This decrease in NF sensitivity and the increased sensitivity to PF were hypothesized to manifest antidepressant-induced, positive, information-processing biases, similar to those reported previously in humans following acute antidepressant treatment (Arnone et al., 2009; Rawlings et al., 2010; Komulainen et al., 2016). Building off these prospective findings, in the current study, we tested a hypothesis that the basal valence of individuals’ sensitivity to feedback, measured as a stable and enduring behavioral trait, could moderate the effects of these two antidepressant drugs on hedonic processing in rats.

The fact that the results of the conducted experiments did not confirm this hypothesis, at least concerning the acute effects of antidepressants on sucrose preference, might suggest various effects of these drugs on the “wanting” and “liking” of rewards. Indeed, according to incentive sensitivity theory, brain mechanisms that determine how much a reward is “wanted” are separate from those that determine how much the reward is “liked” (Berridge and Robinson, 1998). “Wanting,” which was expressed herein by the ratio of win-stay behaviors, is generated by the mesolimbic dopamine system, while “liking,” or the actual pleasurable impact of reward consumption, which is indexed herein by the sucrose preference, is mediated by other, dopamine-independent and mainly opioidergic mechanisms (Berridge and Robinson, 1998). Although they were not investigated in our study, these various mechanisms could contribute to the observed differences in the effects of mirtazapine on win-stay behaviors in the PRL test, as previously observed by Drozd et al. (2019), and the effects on sucrose preference, as reported in the present study.

There are several limitations to this study that need to be mentioned. When considered in the context of depression, the first limitation would be the use of naïve rats. According to Beck (2008) and Harmer et al. (2003b), altered processing of information may play an important role in the effectiveness of antidepressant treatment; however, as mentioned above, a truly naturalistic animal model would require the use of environmental triggers, e.g., stress. Based on the present results, we cannot exclude that the expected interaction between trait sensitivity to feedback and the effects of antidepressants on hedonic processing in the SP test would become more salient if investigated in a model of depressive-like symptoms based on chronic psychosocial stress (Rygula et al., 2005). This limitation, however, in our opinion, does not undermine the validity of the present data, since the antidepressant efficacy of drugs is being widely and commonly studied in naïve animals [e.g., in the forced swim test (Porsolt et al., 2001)] and because both antidepressant drugs tested in the present study have been demonstrated previously to produce “antidepressive-like” effects in naïve animals (Stuart et al., 2013; Drozd et al., 2019).

The second limitation would be the use of only male subjects. Indeed, since the prevalence of the depressive disorder is significantly higher in women than in men, it seems more accurate to investigate the associated processes in females. However, the decision to use only male subjects was based on practical reasons: males do not have an oestrous cycle that could quite likely, by itself, affect the sensitivity to feedback. Thus, to avoid this additional confounding factor, we decided to test only male rats.

The third limitation would be the use of only one method for the measurement of the hedonic capacity of rats. Although indeed testing the hedonic capacity in a variety of other tests e.g., the cookie test (Surget et al., 2011) or the sweet drive test (Mateus-Pinheiro et al., 2014) could make the results more robust, we are convinced that the results obtained in the SP test are valid and reliable. The advantages of this test, which explain its popularity in laboratories throughout the world, are its simplicity and reliability. The method itself has been used in our laboratory for several years and has been thoroughly validated using various behavioral and pharmacological manipulations (Rygula et al., 2005, 2006, 2008, 2013).

Finally, it might be interesting to test the impact of trait sensitivity to feedback on hedonic processing in animals subjected to chronic antidepressant treatment. Although the tested compounds were previously reported to be effective in changing sensitivity to feedback following a single administration (Stuart et al., 2013; Drozd et al., 2019), the full antidepressant effects of these compounds (including their effects on hedonic processing) could perhaps be achieved following prolonged treatment.

The results of our study add to the growing body of experimental data regarding the role of cognitive traits in the development, maintenance, and treatment of affective disorders. These results also show that the immediate effects of some antidepressant drugs on cognitive processing are not immediately conveyed by changes in the hedonic processing of rewards.

## Data Availability Statement

The raw data supporting the conclusions of this article will be made available by the authors, without undue reservation.

## Ethics Statement

All experiments were conducted following the European Union guidelines for the care and use of laboratory animals (2010/63/EU). Experimental protocols were reviewed and approved by the 2nd Local Institutional Animal Care and Use Committee at the Maj Institute of Pharmacology Polish Academy of Sciences in Krakow (Permission no. 242/2017).

## Author Contributions

RR designed the research. PS and KN performed the research. PS, KN, and RR analyzed the data. PS, KN, and RR wrote the article.

## Conflict of Interest

The authors declare that the research was conducted in the absence of any commercial or financial relationships that could be construed as a potential conflict of interest.

## References

[B1] Anderson M. H. Munafo M. R. Robinson E. S. (2013). Investigating the psychopharmacology of cognitive affective bias in rats using an affective tone discrimination task. Psychopharmacology 226, 601–613. 10.1007/s00213-012-2932-5 23239131

[B2] Arnone D. Horder J. Cowen P. J. Harmer C. J. (2009). Early effects of mirtazapine on emotional processing. Psychopharmacology 203, 685–691. 10.1007/s00213-008-1410-6 19031070

[B3] Bari A. Theobald D. E. Caprioli D. Mar A. C. Aidoo-Micah A. Dalley J. W.. (2010). Serotonin modulates sensitivity to reward and negative feedback in a probabilistic reversal learning task in rats. Neuropsychopharmacology 35, 1290–1301. 10.1038/npp.2009.233 20107431 PMC3055347

[B4] Beats B. C. Sahakian B. J. Levy R. (1996). Cognitive performance in tests sensitive to frontal lobe dysfunction in the elderly depressed. Psychol. Med. 26, 591–603. 10.1017/s0033291700035662 8733217

[B5] Beck A. T. (1987). Cognitive models of depression. J. Cogn. Psychother. 1, 5–37.

[B6] Beck A. T. (2008). The evolution of the cognitive model of depression and its neurobiological correlates. Am. J. Psychiatry 165, 969–977. 10.1176/appi.ajp.2008.08050721 18628348

[B7] Belzung C. Willner P. Philippot P. (2015). Depression: from psychopathology to pathophysiology. Curr. Opin. Neurobiol. 30, 24–30. 10.1016/j.conb.2014.08.013 25218233

[B8] Berendsen H. H. Broekkamp C. L. (1997). Indirect *in vivo* 5-HT1A-agonistic effects of the new antidepressant mirtazapine. Psychopharmacology 133, 275–282. 10.1007/s002130050402 9361334

[B9] Berridge K. C. Robinson T. E. (1998). What is the role of dopamine in reward: hedonic impact, reward learning, or incentive salience? Brain Res. Rev. 28, 309–369. 10.1016/s0165-0173(98)00019-8 9858756

[B10] Browning M. Reid C. Cowen P. J. Goodwin G. M. Harmer C. J. (2007). A single dose of citalopram increases fear recognition in healthy subjects. J. Psychopharmacol. 21, 684–690. 10.1177/0269881106074062 17259206

[B11] Clark L. Chamberlain S. R. Sahakian B. J. (2009). Neurocognitive mechanisms in depression: implications for treatment. Annu. Rev. Neurosci. 32, 57–74. 10.1146/annurev.neuro.31.060407.125618 19400725

[B12] de Boer T. (1995). The effects of mirtazapine on central noradrenergic and serotonergic neurotransmission. Int. Clin. Psychopharmacol. 10, 19–23. 10.1097/00004850-199512004-00004 8930006

[B13] Drozd R. Rojek-Sito K. Rygula R. (2017). The trait ‘pessimism’ does not interact with cognitive flexibility but makes rats more vulnerable to stress-induced motivational deficits: results from the attentional set-shifting task. Behav. Brain Res. 335, 199–207. 10.1016/j.bbr.2017.08.028 28842268

[B14] Drozd R. Rychlik M. Fijalkowska A. Rygula R. (2019). Effects of cognitive judgement bias and acute antidepressant treatment on sensitivity to feedback and cognitive flexibility in the rat version of the probabilistic reversal-learning test. Behav. Brain Res. 359, 619–629. 10.1016/j.bbr.2018.10.003 30292902

[B15] Elliott R. Sahakian B. J. Michael A. Paykel E. S. Dolan R. J. (1998). Abnormal neural response to feedback on planning and guessing tasks in patients with unipolar depression. Psychol. Med. 28, 559–571. 10.1017/s0033291798006709 9626713

[B16] Enkel T. Gholizadeh D. von Bohlen Und Halbach O. Sanchis-Segura C. Hurlemann R. Spanagel R.. (2010). Ambiguous-cue interpretation is biased under stress- and depression-like states in rats. Neuropsychopharmacology 35, 1008–1015. 10.1038/npp.2009.204 20043002 PMC3055368

[B17] Golebiowska J. Rygula R. (2017). Effects of acute dopaminergic and serotonergic manipulations in the ACI paradigm depend on the basal valence of cognitive judgement bias in rats. Behav. Brain Res. 327, 133–143. 10.1016/j.bbr.2017.02.013 28212943

[B18] Gosling S. D. (2001). From mice to men: what can we learn about personality from animal research? Psychol. Bull. 127, 45–86. 10.1037/0033-2909.127.1.45 11271756

[B19] Harmer C. J. Bhagwagar Z. Shelley N. Cowen P. J. (2003a). Contrasting effects of citalopram and reboxetine on waking salivary cortisol. Psychopharmacology 167, 112–114. 10.1007/s00213-003-1417-y 12605289

[B21] Harmer C. J. Hill S. A. Taylor M. J. Cowen P. J. Goodwin G. M. (2003b). Toward a neuropsychological theory of antidepressant drug action: increase in positive emotional bias after potentiation of norepinephrine activity. Am. J. Psychiatry 160, 990–992. 10.1176/appi.ajp.160.5.990 12727705

[B20] Harmer C. J. Goodwin G. M. Cowen P. J. (2009). Why do antidepressants take so long to work? A cognitive neuropsychological model of antidepressant drug action. Br. J. Psychiatry 195, 102–108. 10.1192/bjp.bp.108.051193 19648538

[B22] He J. Y. Fang P. Zheng X. Wang C. C. Liu T. H. Zhang B. W.. (2018). Inhibitory effect of celecoxib on agomelatine metabolism *in vitro* and *in vivo*. Drug Des. Dev. Ther. 12, 513–519. 10.2147/dddt.s160316 29563776 PMC5849912

[B23] Howell D. C. (1997). Statistical Methods for Psychology. 4th Edn. Belmont, CA: Duxbury Press.

[B24] Komulainen E. Heikkila R. Meskanen K. Raij T. T. Nummenmaa L. Lahti J.. (2016). A single dose of mirtazapine attenuates neural responses to self-referential processing. J. Psychopharmacol. 30, 23–32. 10.1177/0269881115616384 26577062

[B25] Mateus-Pinheiro A. Patricio P. Alves N. D. Machado-Santos A. R. Morais M. Bessa J. M.. (2014). The sweet drive test: refining phenotypic characterization of anhedonic behavior in rodents. Front. Behav. Neurosci. 8:74. 10.3389/fnbeh.2014.00074 24639637 PMC3945942

[B26] Millan M. J. Gobert A. Lejeune F. Dekeyne A. Newman-Tancredi A. Pasteau V.. (2003). The novel melatonin agonist agomelatine (S20098) is an antagonist at 5-hydroxytryptamine2C receptors, blockade of which enhances the activity of frontocortical dopaminergic and adrenergic pathways. J. Pharmacol. Exp. Ther. 306, 954–964. 10.1124/jpet.103.051797 12750432

[B27] Noworyta-Sokolowska K. Kozub A. Jablonska J. Rodriguez Parkitna J. Drozd R. Rygula R. (2019). Sensitivity to negative and positive feedback as a stable and enduring behavioural trait in rats. Psychopharmacology 236, 2389–2403. 10.1007/s00213-019-05333-w 31375849 PMC6695373

[B28] Papp M. Willner P. Muscat R. (1991). An animal model of anhedonia: attenuation of sucrose consumption and place preference conditioning by chronic unpredictable mild stress. Psychopharmacology 104, 255–259. 10.1007/bf02244188 1876670

[B29] Porsolt R. D. Brossard G. Hautbois C. Roux S. (2001). Rodent models of depression: forced swimming and tail suspension behavioral despair tests in rats and mice. Curr. Protoc. Neurosci. Chapter 8:Unit 8.10A. 10.1002/0471142301.ns0810as14 18428536

[B30] Rawlings N. B. Norbury R. Cowen P. J. Harmer C. J. (2010). A single dose of mirtazapine modulates neural responses to emotional faces in healthy people. Psychopharmacology 212, 625–634. 10.1007/s00213-010-1983-8 20809213

[B31] Rouini M. R. Lavasani H. Sheikholeslami B. Owen H. Giorgi M. (2014). Pharmacokinetics of mirtazapine and its main metabolites after single intravenous and oral administrations in rats at two dose rates. Daru 22:13. 10.1186/2008-2231-22-13 24397986 PMC3896718

[B32] Rychlik M. Bollen E. Rygula R. (2017). Ketamine decreases sensitivity of male rats to misleading negative feedback in a probabilistic reversal-learning task. Psychopharmacology 234, 613–620. 10.1007/s00213-016-4497-1 27933365 PMC5263208

[B33] Rygula R. Abumaria N. Flügge G. Fuchs E. Ruther E. Havemann-Reinecke U. (2005). Anhedonia and motivational deficits in rats: impact of chronic social stress. Behav. Brain Res. 162, 127–134. 10.1016/j.bbr.2005.03.009 15922073

[B34] Rygula R. Abumaria N. Flügge G. Hiemke C. Fuchs E. Ruther E.. (2006). Citalopram counteracts depressive-like symptoms evoked by chronic social stress in rats. Behav. Pharmacol. 17, 19–29. 10.1097/01.fbp.0000186631.53851.71 16377960

[B35] Rygula R. Abumaria N. Havemann-Reinecke U. Ruther E. Hiemke C. Zernig G.. (2008). Pharmacological validation of a chronic social stress model of depression in rats: effects of reboxetine, haloperidol and diazepam. Behav. Pharmacol. 19, 183–196. 10.1097/fbp.0b013e3282fe8871 18469536

[B36] Rygula R. Noworyta-Sokolowska K. Drozd R. Kozub A. (2018). Using rodents to model abnormal sensitivity to feedback in depression. Neurosci. Biobehav. Rev. 95, 336–346. 10.1016/j.neubiorev.2018.10.008 30347197

[B37] Rygula R. Papciak J. Popik P. (2013). Trait pessimism predicts vulnerability to stress-induced anhedonia in rats. Neuropsychopharmacology 38, 2188–2196. 10.1038/npp.2013.116 23660704 PMC3773668

[B38] Rygula R. Papciak J. Popik P. (2014). The effects of acute pharmacological stimulation of the 5-HT, NA and DA systems on the cognitive judgement bias of rats in the ambiguous-cue interpretation paradigm. Eur. Neuropsychopharmacol. 24, 1103–1111. 10.1016/j.euroneuro.2014.01.012 24503278

[B39] Rygula R. Popik P. (2016). Trait “pessimism” is associated with increased sensitivity to negative feedback in rats. Cogn. Affect. Behav. Neurosci. 16, 516–526. 10.3758/s13415-016-0410-y 26902303

[B40] Stuart S. A. Butler P. Munafò M. R. Nutt D. J. Robinson E. S. (2013). A translational rodent assay of affective biases in depression and antidepressant therapy. Neuropsychopharmacology 38, 1625–1635. 10.1038/npp.2013.69 23503126 PMC3717539

[B41] Stuart S. A. Butler P. Munafo M. R. Nutt D. J. Robinson E. S. (2015). Distinct neuropsychological mechanisms may explain delayed- versus rapid-onset antidepressant efficacy. Neuropsychopharmacology 40, 2165–2174. 10.1038/npp.2015.59 25740288 PMC4487826

[B42] Surget A. Tanti A. Leonardo E. D. Laugeray A. Rainer Q. Touma C.. (2011). Antidepressants recruit new neurons to improve stress response regulation. Mol. Psychiatry 16, 1177–1188. 10.1038/mp.2011.48 21537331 PMC3223314

[B43] Willner P. Muscat R. Papp M. (1992). An animal model of anhedonia. Clin. Neuropharmacol. 15, 550A–551A. 10.1097/00002826-199201001-00286 1498945

[B44] Ying S. W. Rusak B. Delagrange P. Mocaer E. Renard P. Guardiola-Lemaitre B. (1996). Melatonin analogues as agonists and antagonists in the circadian system and other brain areas. Eur. J. Pharmacol. 296, 33–42. 10.1016/0014-2999(95)00684-2 8720474

[B45] Yous S. Andrieux J. Howell H. E. Morgan P. J. Renard P. Pfeiffer B.. (1992). Novel naphthalenic ligands with high affinity for the melatonin receptor. J. Med. Chem. 35, 1484–1486. 10.1021/jm00086a018 1315395

[B46] Zupancic M. Guilleminault C. (2006). Agomelatine—a preliminary review of a new antidepressant. CNS Drugs 20, 981–992. 10.2165/00023210-200620120-00003 17140278

